# Potential molecular characteristics in situ in response to repetitive UVB irradiation

**DOI:** 10.1186/s13000-016-0579-y

**Published:** 2016-11-10

**Authors:** Wenqi Chen, Jinhai Zhang

**Affiliations:** 1 Department of Dermatology, Nanjing First Hospital, Nanjing Medical University, 68 Changle Road, Nanjing, Jiangsu 210006 China; 2 Department of Epidemiology, Research Institute for Medicine of Nanjing Command, Nanjing, China

**Keywords:** Molecular characteristics, In situ, Repetitive UVB irradiation, Re-analysis of microarray data, Bioinformatics methods

## Abstract

**Background:**

To identify molecular characteristics in situ in response to repetitive UVB (ultraviolet-B) irradiation.

**Methods:**

Microarray data from the Gene Expression Omnibus were re-analyzed to identify DEGs (differentially expressed genes) between UVB-irradiated and non-irradiated skin biopsies. Enrichment and annotation analyses were performed respectively using DAVID, and TSGene and TAG databases. PPIs (protein-protein interactions) were analyzed using STRING, and miRNAs (microRNAs) and TFs (transcription factors) were predicted separately by miRNA-related databases and ENCODE. Accordingly, the PPI network and regulatory networks were visualized using Cytoscape, and they were merged together to obtain an integrated network for mining densely connected modules.

**Results:**

Altogether, 151 up- and 64 down-regulated genes were identified between UVB-irradiated and non-irradiated skin biopsies, among which down-regulated *DNAJB4* and *SLIT2* were annotated as tumor-suppressors and up-regulated *KIT* was annotated as an oncogene. The up-regulated DEGs were significantly enriched in biological processes related to pigmentation (*DCT*, *SOX10*, *TYRP1*, *TYR*, *MLPH*, *KIT* and *GPR143*), while the down-regulated DEGs were dramatically related to haemopoiesis and the immune system (*GPR183*, *INHBA*, *PTPRC*, *PLEK*, *CD8A* and *IKZF1*). Furthermore, many miRNAs were screened for the DEGs, including miR-206 and miR-496 targeting *KIT*, miR-184 targeting *DCT*, and highly significant miR-337-5p, miR-21 and miR-16. Additionally, TFs were identified for the DEGs, among which *PAX5* and *HNF4A* targeted *MLPH* and *GPR143*, respectively, while *BATF*, *SPI1* and *EP300* jointly target *GPR183*, *PTPRC* and *PLEK*.

**Conclusions:**

The pigmentation and immune system implicated by DEGs, miRNAs and TFs might be important molecular mechanisms in response to UVB irradiation.

## Background

Sunlight is essential for human life, but a previous study has revealed that it may induce 2 million new cases of non-melanoma skin cancer every year in the United States [1]. UV (ultraviolet) may impair the ability of basal keratinocytes to remove melanin and thus lead to hyperpigmentation [2]. Furthermore, UV radiation might be the major contributor to the risk of certain types of skin cancers in UV-radiated outdoor workers, such as basal cell carcinoma and squamous cell carcinoma [3]. UVB (ultraviolet-B) is a type of UV with wavelength ranging from 280 to 320 nm, and excessive UVB irradiation from sunlight may induce oxidative damage and inflammation in skin that further causes sunburn, photo-aging and various skin cancers [4].

Numerous researchers have been exploring the molecular characteristics in response to UVB exposure using animal models or cell culture systems. The activity of tyrosinase essential for melanogenic cascade can be stimulated by UVB exposure that further increases melanin synthesis required for UVB-induced delayed tanning [5]. The membrane-bound stem cell factor is up-regulated and then activates neighboring melanocytes through *kit* receptors in the course of UVB-caused pigmentation [6]. *Nrf2* (nuclear factor, erythroid 2-like 2) may exert protective effects on an inflammation and a sunburn reaction that were induced by UVB exposure via inhibiting inflammation and extracellular matrix degradation [7]. More dangerously, UVB exposure can down-regulate the tumor suppressor *TGFβ* (Transforming growth factor β) signaling, suggesting its oncogenic effects [8]. Additionally, miRNAs (microRNAs) and TFs (transcription factors) are also implicated in the UVB irradiation-induced cellular response via regulating target genes [9, 10].

Although those previous studies have facilitated our understanding of molecular mechanisms in response to UVB exposure, they might not precisely reveal the molecular characteristics in situ where human skin is repetitively irradiated by UVB. Wonseon Choi et al. have demonstrated that UVB irradiation in situ can induce a large number of significant UVB-responsive genes implicated in regulating skin pigmentation, which has a different molecular characterization in response to UVA (ultraviolet-A) exposure [11]. However valuable, the above research focuses on the different molecular characteristics of human skin pigmentation under various types of UV irradiation. It is well known that different analysis procedures can obtain different findings. From a distinct perspective, this study was designed to explore the underlying molecular mechanisms in situ in response to UVB irradiation using various bioinformatics methods.

This study re-analyzed the published microarray data [11] to screen out DEGs (differentially expressed genes) between UVB-irradiated and non-irradiated skin biopsies, which were then subjected to enrichment analyses to identify biological metabolism and annotation analysis to identify tumor-associated DEGs. Then, PPIs (protein-protein interactions) among the DEGs were screened out to construct a PPI network, whilst TFs and miRNAs were screened out to construct regulatory networks. Accordingly, an integrated network among TFs, miRNAs and DEGs was constructed and further mined to identify densely connected modules.

## Methods

*This article does not contain any studies with human participants or animals performed by any of the authors.*

### Gene expression profiles

To obtain a global view of the gene expression patterns induced by exposure to UVB, gene expression profiling data [11] from UVB-irradiated skin biopsies and matched controls were downloaded from the GEO (Gene Expression Omnibus, http://www.ncbi.nlm.nih.gov/geo/) database [12]. The skin biopsies were obtained from six volunteers with Fitzpatrick skin type II–III, whose average age was 37.3 ± 15.7 years. All donors provided written informed consent before enrollment in the irradiation experiment, which was conducted in accordance with the Helsinki guidelines and approved by the Research Involving Human Subjects Committees of Beiersdorf AG. For UVB irradiation, the backs of all participants were irradiated under 290–320 nm for two weeks with a frequency of five times per week using a custom-made filter combination (Tafelmayer, Rosenheim, Germany). Then, UVB-irradiated skin biopsies were obtained 3 days after the last irradiation, and corresponding non-irradiated skin biopsies were taken from comparable skin regions as controls. Then, total RNA was extracted from all skin biopsies to perform single-colour hybridization on an Agilent-014850 Whole Human Genome Microarray 4x44K G4112F.

### Raw data preprocessing and differential expression analysis

The downloaded raw expression profiles were preprocessed through background correction, quantile normalization, probe summarization and transformation of probe IDs to gene symbols by the Limma (Linear Models for Microarray data, version 1.8.18, http://www.bioconductor.org/packages/release/bioc/html/limma.html) package in R [13]. Accordingly, a gene expression matrix was obtained and subsequently subjected to differential expression analysis between UVB-irradiated and non-irradiated skin biopsies using the *t*-test. The p-value of each gene was adjusted by the Benjamini & Hochberg method [14]. Genes with a *P*-value < 0.05 and a |log_2_ fold change (FC)| >0.5 were selected as DEGs. Additionally, in consideration of the complex regulatory effects of TFs and miRNAs on target genes, genes with a *P*-value < 0.01 were identified as expressed genes for subsequent analysis of TFs and miRNAs.

### Enrichment analyses and functional annotations

Functional and pathway enrichment analyses can be implemented using the web-accessible DAVID (Database for Annotation, Visualization and Integrated Discovery, http://david.abcc.ncifcrf.gov/) v6.7 [15], which augments the biological meaning of genome-scale datasets by providing GO (Gene Ontology, version 1.2, http://www.geneontology.org/) [16] functional and KEGG (Kyoto Encyclopedia of Genes and Genomes, version 58, http://www.genome.jp/kegg/pathway.html) [17] pathway enrichment analyses. To interpret the biological meanings of DEGs, GO and KEGG enrichment analyses were respectively implemented for the significantly up-regulated and down-regulated genes, respectively, with all genes in the human genome being used as background. The thresholds were *P*-values < 0.05 and the numbers of enriched genes in each term >2. Additionally, tumor-associated genes were screened separately from the DEGs using TSGene (Tumor Suppressor Gene, http://bioinfo.mc.vanderbilt.edu/TSGene/) (20 March, 2015) [18] and TAG (Tumor Associated Gene, http://www.binfo.ncku.edu.tw/TAG/GeneDoc.php) (20 March, 2015) databases [19].

### Protein-protein interaction network construction

To further explore interactions among the DEGs, they were inputted into the online database STRING v9.1 (Search Tool for the Retrieval of Interacting Genes, http://string-db.org/) [20]. Subsequently, interaction pairs were screened out based on a combined score threshold >0.4, and they were visualized by constructing a PPI network in Cytoscape v2.6 (http://cytoscape.org/) [21]. The hub nodes, corresponding to DEGs with predominant roles in biological processes [22], were further screened from the PPI network.

### Regulatory network construction

To investigate the up-stream regulatory mechanisms, a total of 6 databases were used to predict miRNAs for the expressed genes with the thresholds of *P*-values < 0.05 and the number of target genes ≥2, including miRanda v1.9, MirTarget2 v2.0, PicTar v4, PITA v6, TargetScan v4.1 and miRecords v3 [23]. Moreover, TFs were screened out among the expressed genes based on the ENCODE (Encyclopedia of DNA Elements, https://www.encodeproject.org/) project consortium 2007 [24] and subsequently subjected to detection of TF activity using the partial least square algorithm [25]. Accordingly, regulatory networks of miRNAs-target genes and TFs-target genes were constructed using Cytoscape v2.6.

### Integrated network construction and module screening

The PPI network and the two regulatory networks were further merged together to obtain one integrated network among TFs, miRNAs and DEGs. Subsequently, the integrated network was mined to screen out densely connected modules with default cutoffs (degree cutoff: 2, node score cutoff: 0.2, K-core: 2, and maximum depth: 100) using MCODE v1.31 (Molecular Complex Detection, http://baderlab.org/Software/MCODE) [26]. Functional enrichment analyses were performed for the densely connected modules to identify their biological relevance using DAVID v6.7.

## Results

### Differentially expressed genes

With cutoffs of *P*-value < 0.05 and |log_2_ FC| >0.5, altogether 215 DEGs including 151 up- and 64 down-regulated genes were identified in UVB-irradiated skin biopsies compared with non-irradiated controls. Moreover, 3958 expressed genes with *P*-value < 0.01 were screened out for subsequent TF and miRNA analyses.

### Enrichment and annotation analysis results

The up- and down-regulated DEGs were inputted into the DAVID database to identify significantly enriched GO functional terms and KEGG pathways, respectively. Accordingly, the up-regulated DEGs were significantly enriched in biological processes related to pigmentation, such as the pigmentation during development (*P*-value = 3.63E-08), pigmentation [*P*-value = 5.78E-06; involving *DCT* (dopachrome tautomerase), *SOX10* (SRY (sex determining region Y)-box 10), *TYRP1* (tyrosinase-related protein 1), *TYR* (tyrosinase), *MLPH* (melanophilin), *KIT* (v-kit Hardy-Zuckerman 4 feline sarcoma viral oncogene homolog) and *GPR143* (G protein-coupled receptor 143)], melanin biosynthetic process (*P*-value = 1.59E-03) and melanin metabolic process (*P*-value = 2.03E-03, involving *DCT*, *TYRP1* and *TYR*) (Table 1).

**Table 1:** The significantly enriched GO_BP terms (top 1) and KEGG pathways of up-regulated DEGs

Term	Count	Gene lists	*P*-Value	Adjusted *P*-value
GO_BP: enrichment Score: 3.4367879252049858				
GO_BP: 0048066 pigmentation during development	7	*DCT, SOX10, TYRP1, TYR, MLPH, KIT, GPR143*	3.63E-08	5.65E-05
GO_BP: 0043473 pigmentation	7	*DCT, SOX10, TYRP1, TYR, MLPH, KIT, GPR143*	5.78E-06	0.009009
GO_BP: 0042438 melanin biosynthetic process	3	*DCT, TYRP1, TYR*	1.59E-03	2.449285
GO_BP: 0006582 melanin metabolic process	3	*DCT, TYRP1, TYR*	2.03E-03	3.123208
GO_BP: 0046148 pigment biosynthetic process	4	*DCT, TYRP1, TYR, GPR143*	3.30E-03	5.019067
GO_BP: 0042440 pigment metabolic process	4	*DCT, TYRP1, TYR, GPR143*	4.96E-03	7.448208
GO_BP: 0030318 melanocyte differentiation	3	*SOX10, TYRP1, MLPH*	5.02E-03	7.534839
GO_BP: 0050931 pigment cell differentiation	3	*SOX10, TYRP1, MLPH*	5.76E-03	8.604376
KEGG				
hsa04080: Neuroactive ligand-receptor interaction	6	*SSTR4, GPR156, KISS1R, OPRL1, NPBWR1, GRIN1*	1.58E-02	13.80265
hsa04916: Melanogenesis	4	*DCT, TYRP1, TYR, KIT*	1.99E-02	17.11413
hsa00350: Tyrosine metabolism	3	*DCT, TYRP1, TYR*	2.73E-02	22.81886

Note: *GO* gene ontology, *BP* biological process, *KEGG* Kyoto encyclopedia of genes and genomes, *DEGs* differentially expressed genes. Count, the number of DEGs enriched in the corresponding term. The gene symbols were listed in accordance with the Gene database at NCBI (National Center for Biotechnology Information, http://www.ncbi.nlm.nih.gov/gene/?term)

On the other hand, the down-regulated DEGs were dramatically related to the haemopoiesis progress and immune system, such as the haemopoiesis (*P*-value = 1.38E-04), haemopoietic or lymphoid organ development (*P*-value = 2.34E-04), immune system development [*P*-value = 3.22E-04; involving *GPR183* (G protein-coupled receptor 143), *INHBA* (inhibin, beta A), *PTPRC* (protein tyrosine phosphatase, receptor type, C), *PLEK* (pleckstrin), *CD8A* (CD8a molecule) and *IKZF1* (IKAROS family zinc finger 1)] (Table 2).

**Table 2:** The significantly enriched GO_BP terms (top 1) and KEGG pathways of down-regulated DEGs

Term	Count	Gene lists	*P*-Value	Adjusted *P-*value
GO_BP: enrichment Score: 2.505066250961214				
GO_BP: 0030097 hemopoiesis	7	*GPR183, INHBA, PTPRC, PLEK, CD8A, IKZF1, FLT3*	1.38E-04	0.218579
GO_BP: 0048534 hemopoietic or lymphoid organ development	7	*GPR183, INHBA, PTPRC, PLEK, CD8A, IKZF1, FLT3*	2.34E-04	0.369612
GO_BP: 0050865 regulation of cell activation	6	*INHBA, PTPRC, PLEK, IKZF1, FLT3, IL1B*	3.05E-04	0.482267
GO_BP: 0002520 immune system development	7	*GPR183, INHBA, PTPRC, PLEK, CD8A, IKZF1, FLT3*	3.22E-04	0.509185
GO_BP: 0030098 lymphocyte differentiation	5	*GPR183, PTPRC, CD8A, IKZF1, FLT3*	4.05E-04	0.638643
GO_BP: 0002521 leukocyte differentiation	5	*GPR183, PTPRC, CD8A, IKZF1, FLT3*	1.00E-03	1.572197
GO_BP: 0045619 regulation of lymphocyte differentiation	4	*INHBA, PTPRC, IKZF1, FLT3*	1.27E-03	1.98708
GO_BP: 0045577 regulation of B cell differentiation	3	*INHBA, PTPRC, FLT3*	1.32E-03	2.064808
GO_BP: 0030217 T cell differentiation	4	*PTPRC, CD8A, IKZF1, FLT3*	1.39E-03	2.173407
GO_BP: 0051249 regulation of lymphocyte activation	5	*INHBA, PTPRC, IKZF1, FLT3, IL1B*	1.57E-03	2.458734
KEGG pathways				
hsa04640: Hematopoietic cell lineage	7	*CD8A, FLT3, CD1C, IL1B, CSF2RA, CD1E, IL1A*	1.18E-06	0.001103
hsa04060: Cytokine-cytokine receptor interaction	8	*INHBA, CCL22, FLT1, IL18RAP, FLT3, IL1B, CSF2RA, IL1A*	7.78E-05	0.072785

Note: *GO* gene ontology, *BP* biological process, *KEGG* Kyoto encyclopedia of genes and genomes, *DEGs* differentially expressed genes. Count, the number of DEGs enriched in the corresponding term. The gene symbols were listed in accordance with the Gene database at NCBI (National Center for Biotechnology Information, http://www.ncbi.nlm.nih.gov/gene/?term)

Additionally, functional annotation analysis revealed that the down-regulated *DNAJB4* (DnaJ (Hsp40) homolog, subfamily B, member 4) and *SLIT2* (slit homologue 2) might function as tumor-suppressors, and the up-regulated *KIT* (v-kit Hardy-Zuckerman 4 feline sarcoma viral oncogene homolog) may function as an oncogene.

### Protein-protein interaction network

**Fig. 1:**
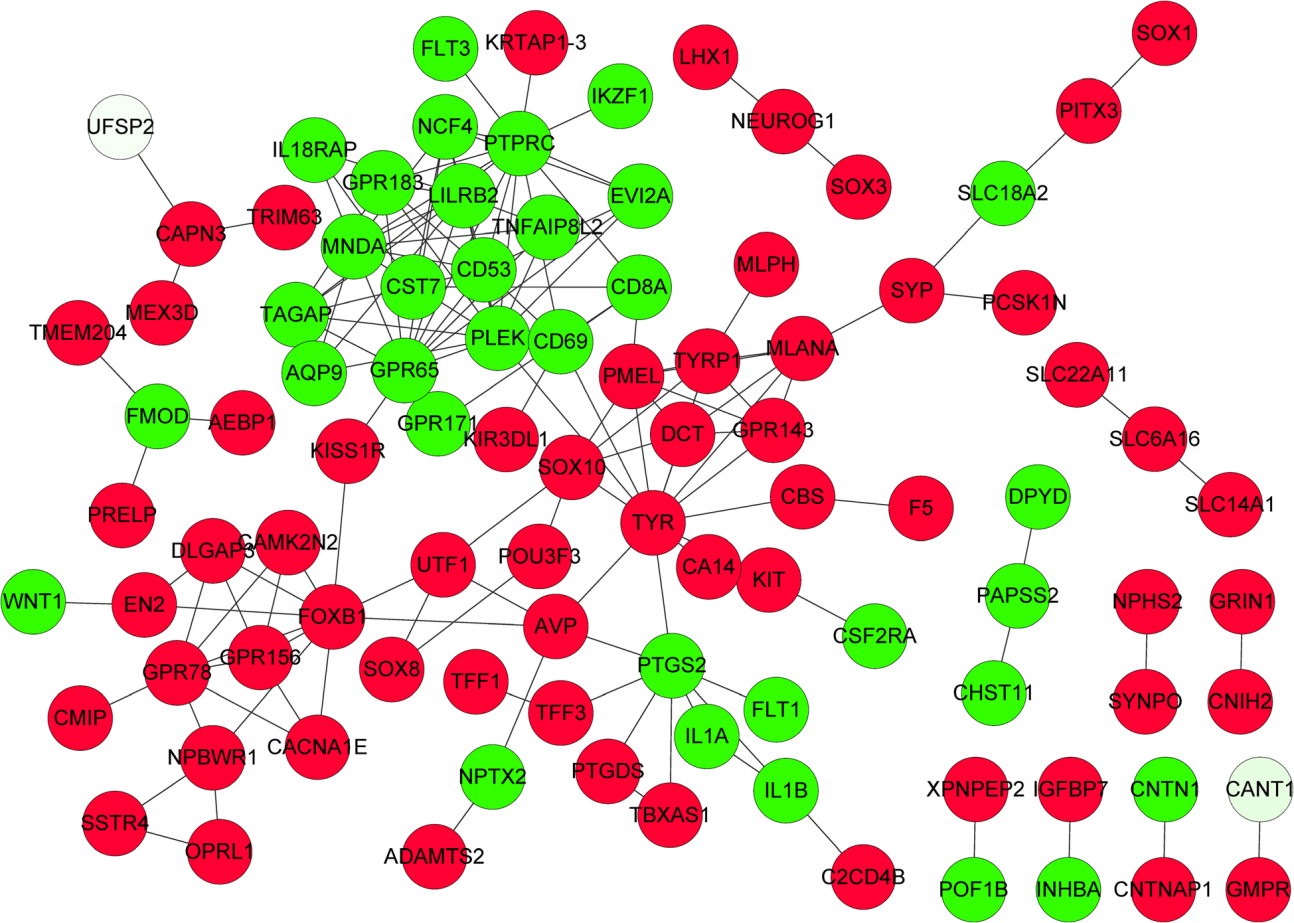
Protein-protein interaction network of differentially expressed genes. *Circular* nodes represent differentially expressed genes and edges represent interactions. *Red* represents up-regulation while *green* represents down-regulation. The color depth represents the statistical significance level

By inputting the DEGs into the STRING database, a total of 152 interaction pairs was screened out and then constructed into a PPI network (Fig. 1). In the network, the top 3 hub nodes with highest connectivity degree were *PTPRC* (degree = 13), *TYR* (degree = 13), and *PLEK* (degree = 12).

### Regulatory networks

**Fig. 2:**
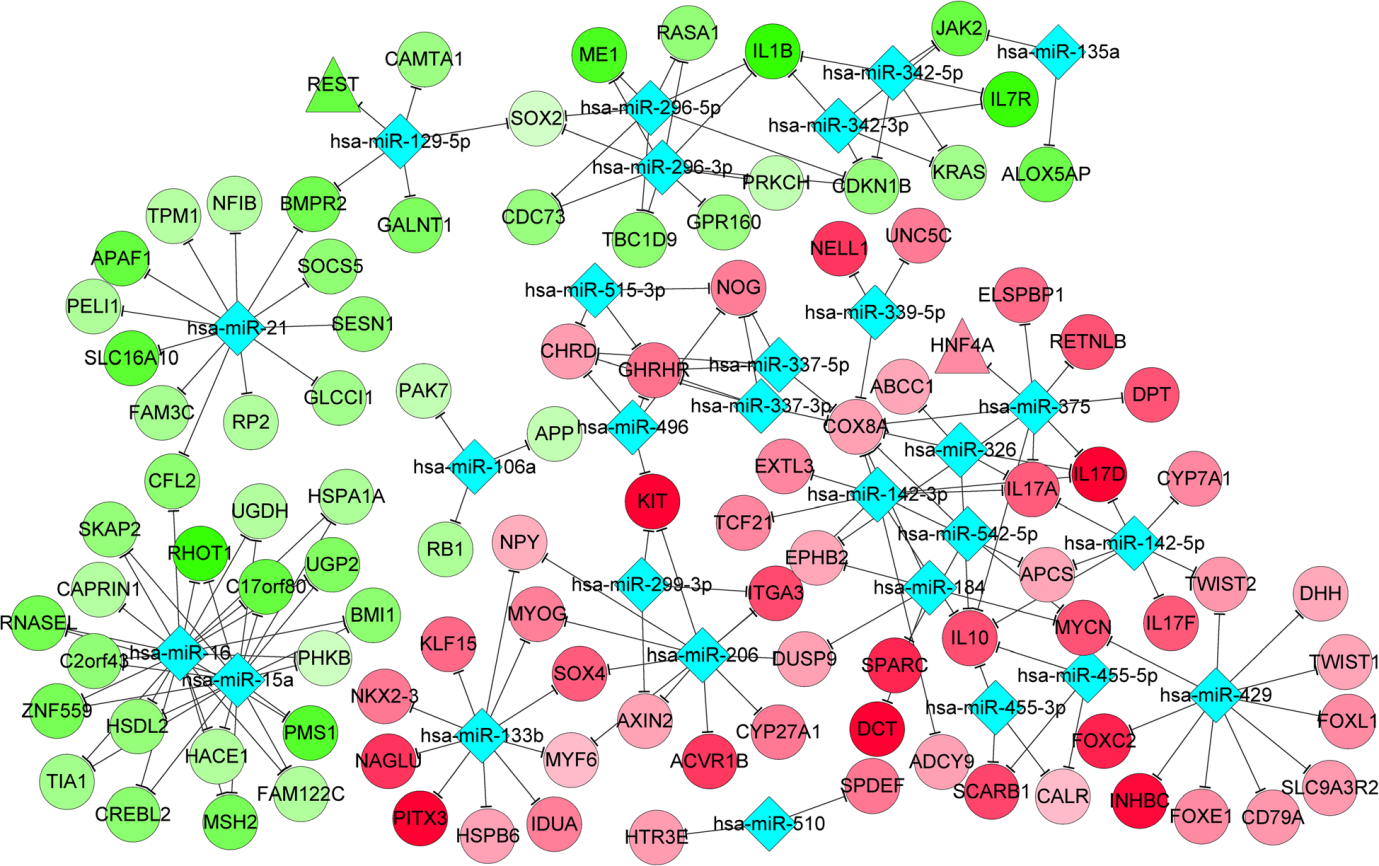
Regulatory network of microRNA-target genes. *Rhombuses* represent microRNAs and *circles* represent differentially expressed genes. *Red* represents up-regulation while *green* represents down-regulation. –—, interactions between DEGs; −—|, regulatory effects of microRNAs on DEGs. DEGs, differentially expressed genes

**Fig. 3:**
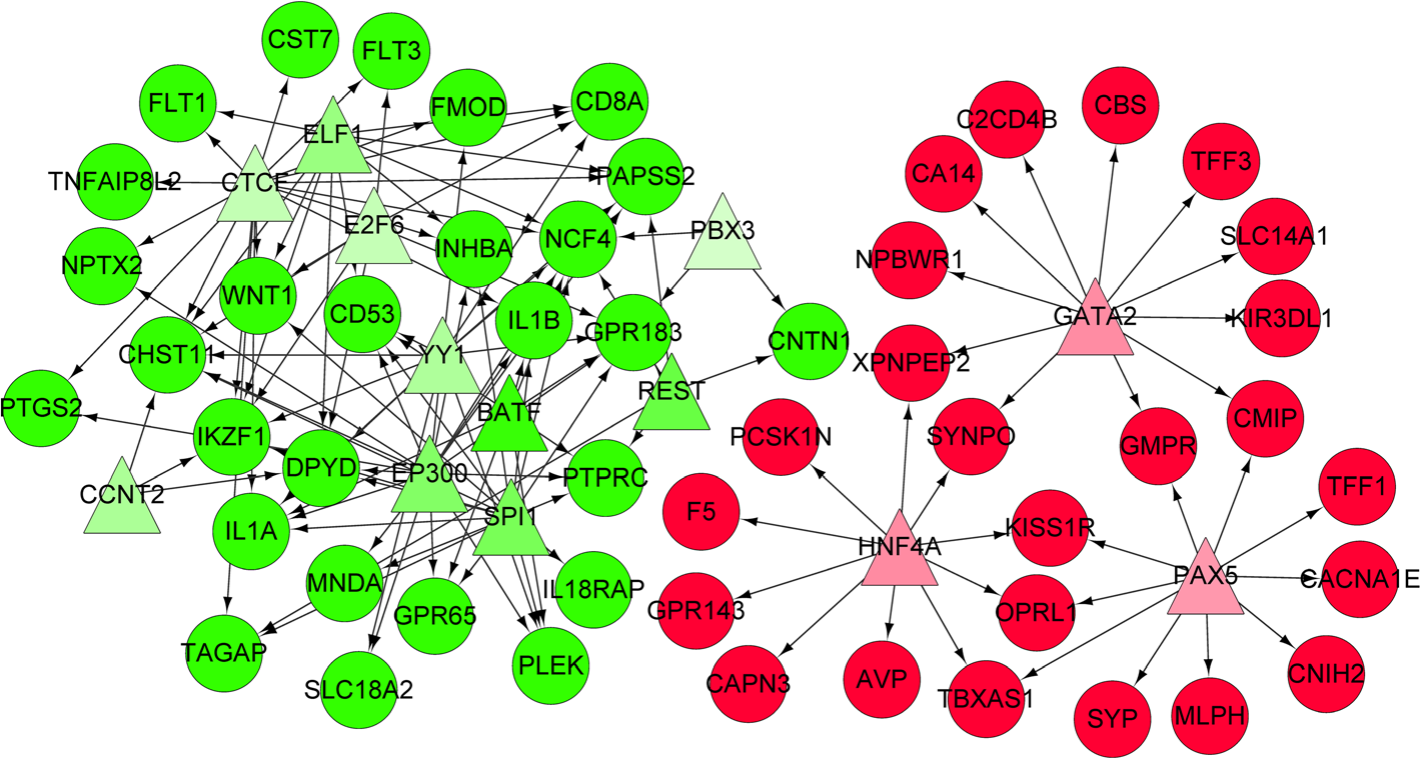
Regulatory network of transcription factors-target genes. *Triangles* represent transcription factors and *circles* represent differentially expressed genes. *Red* represents up-regulation while *green* represents down-regulation. –—, interactions between differentially expressed genes; →, regulatory effects of transcription factors on differentially expressed genes

Furthermore, a number of TFs and miRNAs were screened from the DEGs. From the Fig. 2, significantly up-regulated *KIT* is a potential target of miR-206 and miR-496, and *DCT* may be targeted by miR-184. MiR-337-5p (*P*-value = 7.62E-04) was most significantly enriched for the up-regulated DEGs while miR-21 (*P*-value = 3.36E-04) and miR-16 (*P*-value =8.90E-04) were identified as having the most significance for down-regulated DEGs (Table 3). Moreover, based on Fig. 3, the up-regulated *PAX5* (paired box 5) and *HNF4A* (hepatocyte nuclear factor 4, alpha) targeted *MLPH* and *GPR143*, respectively, while the down-regulated *BATF* (basic leucine zipper transcription factor, ATF-like), *SPI1* (Spi-1 proto-oncogene) and *EP300* (E1A binding protein p300) jointly targeted *GPR183*, *PTPRC* and *PLEK*. The activity differences between UVB-irradiated and non-irradiated skin biopsies were identified as 4.78E-05, 5.24E-02, −1.06E-01, −1.05E-01 and −8.42E-02 for *PAX5*, *HNF4A*, *BATF*, *SPI1* and *EP300*, respectively (Table 4).

**Table 3:** The miRNAs targeting the differentially expressed genes (DEGs)

MicroRNA	Count	*P*-value
Targeting up-regulated DEGs		
hsa-miR-337-5p	4	7.62E-04
hsa-miR-326	5	1.50E-03
hsa-miR-496	4	1.66E-03
hsa-miR-515-3p	3	2.53E-03
hsa-miR-542-5p	3	5.91E-03
hsa-miR-510	2	7.75E-03
hsa-miR-142-3p	9	8.97E-03
hsa-miR-142-5p	7	1.07E-02
hsa-miR-337-3p	4	1.18E-02
hsa-miR-133b	10	1.69E-02
hsa-miR-375	9	1.87E-02
hsa-miR-206	11	1.90E-02
hsa-miR-299-3p	3	2.71E-02
hsa-miR-455-5p	3	2.71E-02
hsa-miR-429	10	3.02E-02
hsa-miR-184	5	3.20E-02
hsa-miR-339-5p	3	3.81E-02
hsa-miR-455-3p	3	3.81E-02
Targeting down-regulated DEGs		
hsa-miR-21	12	3.36E-04
hsa-miR-16	20	8.90E-04
hsa-miR-15a	18	1.55E-03
hsa-miR-296-3p	9	5.86E-03
hsa-miR-106a	3	1.07E-02
hsa-miR-135a	2	2.09E-02
hsa-miR-129-5p	5	4.00E-02
hsa-miR-342-3p	5	4.00E-02
hsa-miR-342-5p	5	4.00E-02
hsa-miR-296-5p	7	4.71E-02

**Table 4:** The activity differences of transcription factors

Transcription factors	Activity difference
Up-regulated	
*NFE2*	3.68E-01
*ZBTB33*	3.28E-01
*NR3C1*	2.82E-01
*HDAC8*	2.34E-01
*SMARCC2*	1.34E-01
*CCNT2*	1.10E-01
*PPARGC1A*	5.60E-02
*HNF4A*	5.24E-02
*GATA2*	2.12E-02
*PAX5*	4.78E-05
Down-regulated	
*BRF1*	−3.95E-01
*CTCF*	−2.34E-01
*YY1*	−1.68E-01
*ESRRA*	−1.48E-01
*BATF*	−1.06E-01
*SPI1*	−1.05E-01
*ELF1*	−9.63E-02
*EP300*	−8.42E-02
*E2F6*	−6.76E-02
*REST*	−1.88E-02
*PBX3*	−1.49E-02

Note: Activity difference = Activity in UVB-irradiated skin biopsies – Activity in non-irradiated skin biopsies

### Integrated network and screened modules

**Fig. 4:**
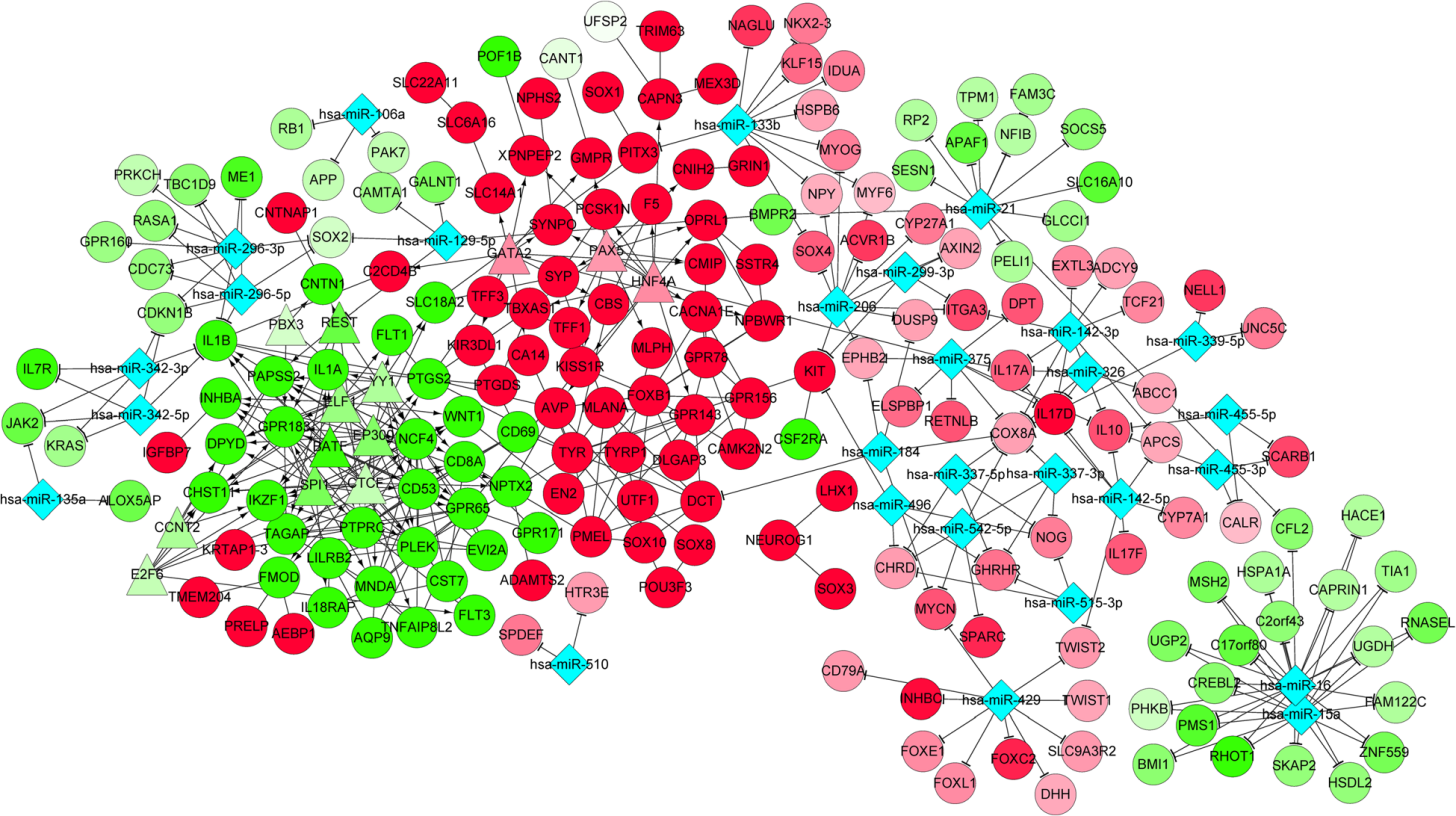
Integrated network among transcription factors, microRNAs and differentially expressed genes. *Rhombuses* represent microRNAs, *triangles* represent transcription factors and circles represent differentially expressed genes. *Red* represents up-regulation while *green* represents down-regulation. –—, interactions between differentially expressed genes; −—|, regulatory effects of microRNAs on differentially expressed genes; →, regulatory effects of transcription factors on differentially expressed genes

**Fig. 5:**
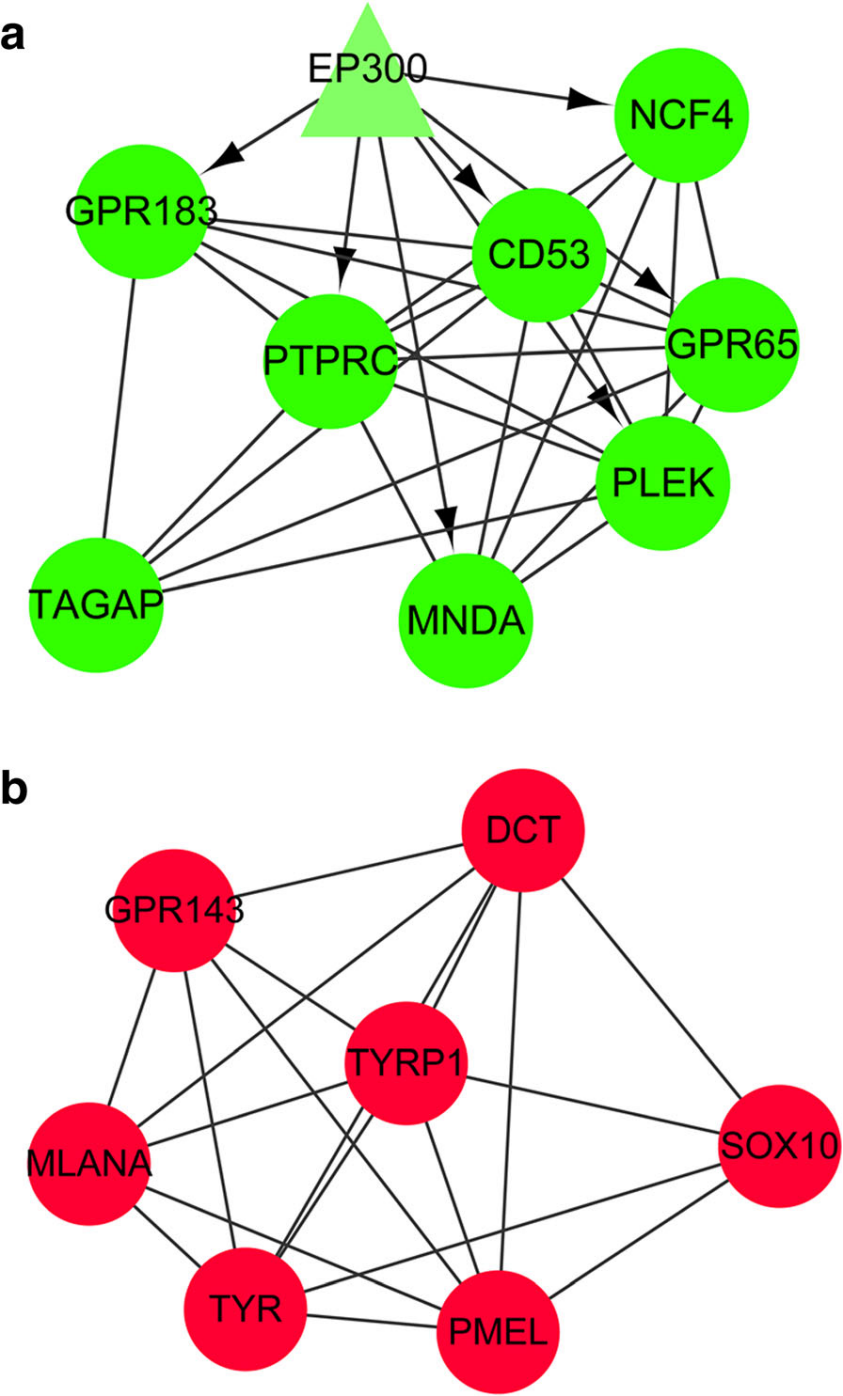
Two densely connected modules screened from the integrated network. **a** down-regulated module; **b** up-regulated module. *Triangles* represent transcription factors and *circles* represent differentially expressed genes. *Red* represents up-regulation while *green* represents down-regulation. –—, interactions between differentially expressed genes; →, regulatory effects of transcription factors on differentially expressed genes

By merging the PPI network and two regulatory networks, an integrated network among TFs, miRNAs and DEGs was obtained (Fig. 4), which was then mined to obtain two modules with highest score (Fig. 5). In the first module, there were 9 down-regulated DEGs including one TF, *EP300*, which is significantly associated with the immune system, such as the immune response (involving *GPR183* and *PTPRC*), haemopoiesis, haemopoietic or lymphoid organ development, immune system development and cell activation (involving *GPR183*, *PTPRC* and *PLEK*) (Table 3). In contrast, there were 7 up-regulated DEGs in the second module, which are dramatically related to pigmentation, including the pigmentation during development, pigmentation, pigment biosynthetic process and pigment metabolic process (involving *DCT*, *TYRP1*, *TYR* and *GPR143*) (Table 5).

**Table 5:** The top 5 significantly enriched GO_BP terms of screened modules

Term	Count	Gene lists	*P*-Value	Adjusted *P*-value
Module 1 Score: 7.75				
GO_BP: 0006955 ~ immune response	4	*GPR183, PTPRC, NCF4, GPR65*	2.35E-03	3.251619
GO_BP: 0030097 ~ hemopoiesis	3	*GPR183, PTPRC, PLEK*	4.34E-03	5.921337
GO_BP: 0048534 ~ hemopoietic or lymphoid organ development	3	*GPR183, PTPRC, PLEK*	5.25E-03	7.113787
GO_BP: 0002520 ~ immune system development	3	*GPR183, PTPRC, PLEK*	5.89E-03	7.959213
GO_BP: 0001775 ~ cell activation	3	*GPR183, PTPRC, PLEK*	6.36E-03	8.562876
Module 2 Score: 6.333				
GO_BP: 0048066 ~ pigmentation during development	5	*DCT, SOX10, TYRP1, TYR, GPR143*	1.07E-11	1.10E-08
GO_BP: 0043473 ~ pigmentation	5	*DCT, SOX10, TYRP1, TYR, GPR143*	3.26E-10	3.35E-07
GO_BP: 0046148 ~ pigment biosynthetic process	4	*DCT, TYRP1, TYR, GPR143*	8.84E-08	9.07E-05
GO_BP: 0042440 ~ pigment metabolic process	4	*DCT, TYRP1, TYR, GPR143*	1.37E-07	1.41E-04
GO_BP: 0042438 ~ melanin biosynthetic process	3	*DCT, TYRP1, TYR*	1.84E-06	0.001882

Note: *GO* gene ontology, *BP* biological process. Count, the number of differentially expressed genes enriched in the corresponding term. The gene symbols were listed in accordance with the Gene database at NCBI (National Center for Biotechnology Information, http://www.ncbi.nlm.nih.gov/gene/?term)

## Discussions

Using expression profiling of UV radiation-induced melanocytes, Yang et al. revealed a set of p53 target genes in response to UV radiation through the Ingenuity® Pathway Analysis program [27]. In 2015, López et al. conducted a time-series analysis to compare the transcriptional profile of dark and light melanocyte lines under basal conditions and after UVB radiation, finding that ribosomal proteins can interact with the p53 signaling pathway in response to UVB [28]. This study identified a total of 151 up-regulated and 64 down-regulated genes in skin in response to UVB irradiation, which probably remained increased after repetitive exposure for approximately 2–3 weeks [11]. The up-regulated DEGs were significantly related to pigmentation, whereas the down-regulated DEGs were dramatically associated with immunity. The functional enrichment results were observed and were similar in the screened modules. As mentioned above, the up-regulated DEGs were significantly enriched in biological processes related to pigmentation. The obtained results are consistent with a previous study [11], implying the reliability of our research. However, the p53 pathway was not enriched in this study, indicating that in vitro and in situ studies have discrepancies and that an in situ study might be more credible. Importantly, this study further identified a number of TFs and miRNAs potentially targeting DEGs.

Several DEGs were enriched in pigmentation, such as *DCT*, *SOX10*, *TYRP1*, *TYR*, *MLPH*, *KIT* and *GPR143*, whose relationships with pigmentation have been revealed extensively in a previous study [11]. However, this study further revealed close interactions among *DCT*, *SOX10*, *TYRP1*, *TYR* and *GPR143* in a densely connected module, suggesting a collaborative stimulating effect on the pigmentation process. Moreover, *TYR* had many interactions with other DEGs and was a hub node in the PPI network, implying predominant roles in the biological processes of pigmentation. Additionally, the up-regulated *KIT*, which was previously identified as a oncogenic drivers in sun-impaired skin, was annotated as a potential oncogene in this study [29].

Notably, some TFs and miRNAs were screened from the up-regulated DEGs. *KIT* is a potential target of miR-206 and miR-496, and *DCT* may be targeted by miR-184. As previously reported, miR-206 acts as a tumor suppressor in melanoma via inhibiting cancer cell growth and migration [30], miR-496 is involved in silencing gene expression in breast cancer, and miR-184 antagonizes miR-205 to protect against stratified squamous epithelia [31]. In consideration of the involvement of *KIT* and *DCT* in pigmentation and carcinogenesis, miRNAs might also be implicated in pigmentation in response to UVB irradiation. Moreover, miR-337, which can have a tumor suppressive effect on pancreatic ductal adenocarcinoma [32], was most significantly enriched, implying its involvement in the cellular response to UVB exposure. On the other hand, *PAX5* and *HNF4A* were identified as two TFs targeting *MLPH* and *GPR143*, respectively. *MLPH* has been demonstrated as a putative pigmentation genes in East Asia [33], and *GPR143* is targeted to melanosomes as a G protein-coupled receptor in pigment cells [34]. Thus, *PAX5* and *HNF4A* might be involved in the regulation of pigmentation under UVB exposure. The up-regulation of DEGs may be associated with the screened the miRNAs and TFs under UVB irradiation.

On the other hand, the down-regulated DEGs were related to the immune system, suggesting an inactive immune regulation, e.g., *GPR183*, *INHBA*, *PTPRC*, *PLEK*, *CD8A* and *IKZF1*. UVB irradiation is responsible for inducing systemic immune suppression, which is an important contributor to skin cancer [35]. The relevance of those DEGs to the immune system or cancer has been revealed. Constitutively activated *PTPRC* modulates neutrophil recruitment and integrin activation during inflammation [36]. *GPR183*, which is primarily expressed in lymphoid cells, is important for the adaptive immune response [37]; as well as *PLEK* and *CD8A* are also important in this response [38, 39]. In addition, *IKZF1* encodes a TF regulating lymphocyte differentiation [40], and *INHBA*, which belongs to the TGF-β superfamily, is associated with several cancers [41]. In the screened module, we further identified close interactions among *GPR183*, *PTPRC* and *PLEK* and observed their enrichment in the immune system. Notably, *PTPRC* and *PLEK* were hub nodes in the PPI network. Therefore, these down-regulated DEGs might jointly contribute to the suppressive immune response to UVB exposure, which might be a cause of skin cancer development. Additionally, the down-regulated *DNAJB4*, which is implicated in human gastric carcinomas [42], and *SLIT2*, which is involved in pancreatic cancer [43], are also annotated as tumor suppressors, suggesting their potential roles in UVB-induced skin cancers.

Additionally, some miRNAs were identified for the down-regulated DEGs, among which miR-21 and miR-16 were the most significantly enriched. The expression of miR-21 could be decreased by UVB irradiation in NIH3T3 cells [44]. MiR-16 targets the thyroid hormone receptor and nuclear factor-kappaB, which are vital for initiating epithelial immune responses [45]. Thus, miR-21 and miR-16 may play a role in the UVB-induced immunosuppressive response via down-regulating immune-related genes. In addition, *GPR183*, *PTPRC* and *PLEK* are all targeted by TFs *BATF*, *SPI1* and *EP300*, which have been demonstrated to be involved in the immune system [46–48]. Notably, we also observed interactions of *EP300* with other down-regulated DEGs in the screened module. The p53/p300 complex can be recruited to the promoters of p53 target genes that are related to cell growth and apoptosis under UVB irradiation [49]. Therefore, the TFs and miRNAs may be involved in the cellular immune response to UVB exposure.

## Conclusion

In conclusion, this study identified 151 up-regulated and 64 down-regulated genes implicated in the pigmentation and immune system response to UVB exposure. UVB-induced pigmentation has been demonstrated in previous studies, but the down-regulation of respectively by UVB exposure involved in the immune system have not been revealed. Furthermore, this study screened some TFs and miRNAs for the DEGs, which might play important roles in UVB irradiation-induced pigmentation and the immunosuppressive response. However, although we identified many DEGs, TFs and miRNAs with potential critical roles in UVB irradiation-induced cellular response, the obtained results relied on bioinformatics methods and need further validation.

## Abbreviations

*BATF*: Basic leucine zipper transcription factor, ATF-like
*CD8A*: CD8a molecule
DAVID: Database for annotation, visualization and integrated discovery
*DCT*: Dopachrome tautomerase
DEGs: Differentially expressed genes
DEGs: Differentially expressed genes
*DNAJB4*: DnaJ (Hsp40) homolog, subfamily B, member 4
ENCODE: Encyclopedia of DNA elements
*EP300*: E1A binding protein p300
GEO: Gene expression omnibus
GO: Gene ontology
*GPR143*: G protein-coupled receptor 143
*GPR183*: G protein-coupled receptor 143
*HNF4A*: Hepatocyte nuclear factor 4, alpha
*IKZF1*: IKAROS family zinc finger 1
*INHBA*: Inhibin, beta A
KEGG: Kyoto encyclopedia of genes and genomes
*KIT*: v-kit Hardy-Zuckerman 4 feline sarcoma viral oncogene homolog
*KIT*: v-kit Hardy-Zuckerman 4 feline sarcoma viral oncogene homolog
MCODE: Molecular complex detection
miRNAs: microRNAs
*MLPH*: Melanophilin
*PAX5*: Paired box 5
*PLEK*: Pleckstrin
PPIs: Protein-protein interactions
*PTPRC*: Protein tyrosine phosphatase, receptor type, C
*SLIT2*: Slit homologue 2
*SOX10*: SRY (sex determining region Y)-box 10
*SPI1*: Spi-1 proto-oncogene
STRING: Search tool for the retrieval of interacting genes
TAG: Tumor associated gene
TFs: Transcription factors
*TGFβ*: Transforming growth factor β
TSGene: Tumor suppressor gene
*TYR*: Tyrosinase
*TYRP1*: Tyrosinase-related protein 1
UV: Ultraviolet
UVA: Ultraviolet-A
UVB: Ultraviolet-B

## References

[1] Rogers HW, Weinstock MA, Harris AR, Hinckley MR, Feldman SR, Fleischer AB, Coldiron BM (2010). Incidence estimate of nonmelanoma skin cancer in the United States, 2006. Arch Dermatol.

[2] Coelho SG, Valencia JC, Yin L, Smuda C, Mahns A, Kolbe L, Miller SA, Beer JZ, Zhang G, Tuma PL, Hearing VJ. UV exposure modulates hemidesmosome plasticity, contributing to long-term pigmentation in human skin. J Pathol. 2014. doi:10.1002/path.4497 10.1002/path.4497 PMC4398603 25488118

[3] Bauer A, Beissert S, Knuschke P (2015). [Prevention of occupational solar UV radiation-induced epithelial skin cancer]. Hautarzt.

[4] Rigo LA, Silva CR, Oliveira SM, Cabreira TN, Silva CB, Ferreira J, Beck RC. Nanoencapsulation of rice bran oil increases its protective effects against UVB radiation-induced skin injury in mice. European journal of pharmaceutics and biopharmaceutics : official journal of Arbeitsgemeinschaft fur Pharmazeutische Verfahrenstechnik eV. 2015. doi:10.1016/j.ejpb.2015.03.020 10.1016/j.ejpb.2015.03.020 25818120

[5] An SM, Koh JS, Boo YC (2010). p-coumaric acid not only inhibits human tyrosinase activity in vitro but also melanogenesis in cells exposed to UVB. Phytother Res.

[6] Kasamatsu S, Hachiya A, Shimotoyodome Y, Kameyama A, Miyauchi Y, Higuchi K, Fujimori T, Ohuchi A, Shibuya Y, Kitahara T (2014). The inhibitory effect of a Platycodon root extract on ultraviolet B-induced pigmentation due to a decrease in Kit expression. J Nat Med.

[7] Saw CL, Yang AY, Huang MT, Liu Y, Lee JH, Khor TO, Su ZY, Shu L, Lu Y, Conney AH, Kong AN (2014). Nrf2 null enhances UVB-induced skin inflammation and extracellular matrix damages. Cell Biosci.

[8] Popoca-Cuaya M, Diaz-Chavez J, Hernandez-Monge J, Alvarez-Rios E, Lambert PF, Gariglio P. The HPV16 E6 oncoprotein and UVB irradiation inhibit the tumor suppressor TGFbeta pathway in the epidermis of the K14E6 transgenic mouse. Exp Dermatol. 2015. doi:10.1111/exd.12689 10.1111/exd.12689 25776923

[9] Cha HJ, Kim OY, Lee GT, Lee KS, Lee JH, Park IC, Lee SJ, Kim YR, Ahn KJ, An IS, An S, Bae S (2014). Identification of ultraviolet B radiationinduced microRNAs in normal human dermal papilla cells. Mol Med Rep.

[10] Cooper SJ, Bowden GT (2007). Ultraviolet B regulation of transcription factor families: roles of nuclear factor-kappa B (NF-kappaB) and activator protein-1 (AP-1) in UVB-induced skin carcinogenesis. Curr Cancer Drug Targets.

[11] Choi W, Miyamura Y, Wolber R, Smuda C, Reinhold W, Liu H, Kolbe L, Hearing VJ (2010). Regulation of human skin pigmentation in situ by repetitive UV exposure: molecular characterization of responses to UVA and/or UVB. J Investig Dermatol.

[12] Edgar R, Domrachev M, Lash AE (2002). Gene Expression Omnibus: NCBI gene expression and hybridization array data repository. Nucleic Acids Res.

[13] Smyth GK. Limma: linear models for microarray data. Bioinformatics and computational biology solutions using R and Bioconductor. New York: Springer; 2005. p. 397–420.

[14] Ferreira J, Zwinderman A (2006). On the Benjamini–Hochberg method. Ann Stat.

[15] Dennis G, Sherman BT, Hosack DA, Yang J, Gao W, Lane HC, Lempicki RA (2003). DAVID: database for annotation, visualization, and integrated discovery. Genome Biol.

[16] Ashburner M, Ball CA, Blake JA, Botstein D, Butler H, Cherry JM, Davis AP, Dolinski K, Dwight SS, Eppig JT (2000). Gene Ontology: tool for the unification of biology. Nat Genet.

[17] Kanehisa M, Goto S, Sato Y, Furumichi M, Tanabe M. KEGG for integration and interpretation of large-scale molecular data sets. Nucleic Acids Res. 2012;40:D109-14. 10.1093/nar/gkr988 PMC3245020 22080510

[18] Zhao M, Sun J, Zhao Z (2013). TSGene: a web resource for tumor suppressor genes. Nucleic Acids Res.

[19] Chen JS, Hung WS, Chan HH, Tsai SJ, Sun HS (2013). In silico identification of oncogenic potential of fyn-related kinase in hepatocellular carcinoma. Bioinformatics.

[20] Jensen LJ, Kuhn M, Stark M, Chaffron S, Creevey C, Muller J, Doerks T, Julien P, Roth A, Simonovic M (2009). STRING 8—a global view on proteins and their functional interactions in 630 organisms. Nucleic Acids Res.

[21] Kohl M, Wiese S, Warscheid B. Cytoscape: software for visualization and analysis of biological networks. Data Mining in Proteomics. Methods Mol Biol: Springer; 2011. p. 291–303. 10.1007/978-1-60761-987-1_18 21063955

[22] He X, Zhang J (2006). Why do hubs tend to be essential in protein networks?. PLoS Genet.

[23] Gutiérrez NC, Sarasquete ME, Misiewicz-Krzeminska I, Delgado M, De Las Rivas J, Ticona F, Ferminan E, Martin-Jimenez P, Chillon C, Risueno A (2010). Deregulation of microRNA expression in the different genetic subtypes of multiple myeloma and correlation with gene expression profiling. Leukemia.

[24] Raney BJ, Cline MS, Rosenbloom KR, Dreszer TR, Learned K, Barber GP, Meyer LR, Sloan CA, Malladi VS, Roskin KM, Suh BB, Hinrichs AS, Clawson H, Zweig AS, Kirkup V, Fujita PA, Rhead B, Smith KE, Pohl A, Kuhn RM, Karolchik D, Haussler D, Kent WJ (2011). ENCODE whole-genome data in the UCSC genome browser (2011 update). Nucleic Acids Res.

[25] Boulesteix AL, Strimmer K (2005). Predicting transcription factor activities from combined analysis of microarray and ChIP data: a partial least squares approach. Theor Biol Med Model.

[26] Bader GD, Hogue CW (2003). An automated method for finding molecular complexes in large protein interaction networks. BMC Bioinformatics.

[27] Yang G, Zhang G, Pittelkow MR, Ramoni M, Tsao H (2006). Expression Profiling of UVB Response in Melanocytes Identifies a Set of p53-Target Genes. J Investig Dermatol.

[28] López S, Smithzubiaga I, Garcia de Galdeano A, Boyano MD, García O, Gardeazábal J, Martinezcadenas C, Izagirre N, De la Rua C, Alonso S (2015). Comparison of the Transcriptional Profiles of Melanocytes from Dark and Light Skinned Individuals under Basal Conditions and Following Ultraviolet-B Irradiation. PLoS One.

[29] Dahl C, Abildgaard C, Riber-Hansen R, Steiniche T, Lade-Keller J, Guldberg P (2015). KIT is a frequent target for epigenetic silencing in cutaneous melanoma. J Invest Dermatol.

[30] Georgantas RW, Streicher K, Luo X, Greenlees L, Zhu W, Liu Z, Brohawn P, Morehouse C, Higgs BW, Richman L, Jallal B, Yao Y, Ranade K (2014). MicroRNA-206 induces G1 arrest in melanoma by inhibition of CDK4 and Cyclin D. Pigment Cell Melanoma Res.

[31] Yu J, Ryan DG, Getsios S, Oliveira-Fernandes M, Fatima A, Lavker RM (2008). MicroRNA-184 antagonizes microRNA-205 to maintain SHIP2 levels in epithelia. Proc Natl Acad Sci U S A.

[32] Zhang R, Leng H, Huang J, Du Y, Wang Y, Zang W, Chen X, Zhao G (2014). miR-337 regulates the proliferation and invasion in pancreatic ductal adenocarcinoma by targeting HOXB7. Diagn Pathol.

[33] Hider JL, Gittelman RM, Shah T, Edwards M, Rosenbloom A, Akey JM, Parra EJ (2013). Exploring signatures of positive selection in pigmentation candidate genes in populations of East Asian ancestry. BMC Evol Biol.

[34] Falletta P, Bagnato P, Bono M, Monticone M, Schiaffino MV, Bennett DC, Goding CR, Tacchetti C, Valetti C (2014). Melanosome-autonomous regulation of size and number: the OA1 receptor sustains PMEL expression. Pigment Cell Melanoma Res.

[35] Chacon-Salinas R, Chen L, Chavez-Blanco AD, Limon-Flores AY, Ma Y, Ullrich SE (2014). An essential role for platelet-activating factor in activating mast cell migration following ultraviolet irradiation. J Leukoc Biol.

[36] Germena G, Volmering S, Sohlbach C, Zarbock A (2015). Mutation in the CD45 inhibitory wedge modulates integrin activation and leukocyte recruitment during inflammation. J Immunol.

[37] Gessier F, Preuss I, Yin H, Rosenkilde MM, Laurent S, Endres R, Chen YA, Marsilje TH, Seuwen K, Nguyen DG, Sailer AW (2014). Identification and characterization of small molecule modulators of the Epstein-Barr virus-induced gene 2 (EBI2) receptor. J Med Chem.

[38] Cremonesi P, Capoferri R, Pisoni G, Del Corvo M, Strozzi F, Rupp R, Caillat H, Modesto P, Moroni P, Williams JL, Castiglioni B, Stella A (2012). Response of the goat mammary gland to infection with Staphylococcus aureus revealed by gene expression profiling in milk somatic and white blood cells. BMC Genomics.

[39] Ghio M, Contini P, Ubezio G, Ansaldi F, Setti M, Tripodi G (2014). Blood transfusions with high levels of contaminating soluble HLA-I correlate with levels of soluble CD8 in recipients’ plasma; a new control factor in soluble HLA-I-mediated transfusion-modulated immunomodulation?. Blood Transfus.

[40] Yoshida T, Georgopoulos K (2014). Ikaros fingers on lymphocyte differentiation. Int J Hematol.

[41] Tang W, Morgan DR, Meyers MO, Dominguez RL, Martinez E, Kakudo K, Kuan PF, Banet N, Muallem H, Woodward K, Speck O, Gulley ML (2012). Epstein-barr virus infected gastric adenocarcinoma expresses latent and lytic viral transcripts and has a distinct human gene expression profile. Infect Agent Cancer.

[42] Simoes-Correia J, Silva DI, Melo S, Figueiredo J, Caldeira J, Pinto MT, Girao H, Pereira P, Seruca R (2014). DNAJB4 molecular chaperone distinguishes WT from mutant E-cadherin, determining their fate in vitro and in vivo. Hum Mol Genet.

[43] Secq V, Leca J, Bressy C, Guillaumond F, Skrobuk P, Nigri J, Lac S, Lavaut MN, Bui TT, Thakur AK, Callizot N, Steinschneider R, Berthezene P, Dusetti N, Ouaissi M, Moutardier V, Calvo E, Bousquet C, Garcia S, Bidaut G, Vasseur S, Iovanna JL, Tomasini R (2015). Stromal SLIT2 impacts on pancreatic cancer-associated neural remodeling. Cell Death Dis.

[44] Guo L, Huang ZX, Chen XW, Deng QK, Yan W, Zhou MJ, Ou CS, Ding ZH (2009). Differential expression profiles of microRNAs in NIH3T3 cells in response to UVB irradiation. Photochem Photobiol.

[45] Zhou R, Li X, Hu G, Gong AY, Drescher KM, Chen XM (2012). miR-16 targets transcriptional corepressor SMRT and modulates NF-kappaB-regulated transactivation of interleukin-8 gene. PLoS One.

[46] Ubel C, Sopel N, Graser A, Hildner K, Reinhardt C, Zimmermann T, Rieker RJ, Maier A, Neurath MF, Murphy KM, Finotto S (2014). The activating protein 1 transcription factor basic leucine zipper transcription factor, ATF-like (BATF), regulates lymphocyte- and mast cell-driven immune responses in the setting of allergic asthma. J Allergy Clin Immunol.

[47] Matulova M, Havlickova H, Sisak F, Babak V, Rychlik I (2013). SPI1 defective mutants of Salmonella enterica induce cross-protective immunity in chickens against challenge with serovars Typhimurium and Enteritidis. Vaccine.

[48] Liu Y, Wang L, Predina J, Han R, Beier UH, Wang LC, Kapoor V, Bhatti TR, Akimova T, Singhal S, Brindle PK, Cole PA, Albelda SM, Hancock WW (2013). Inhibition of p300 impairs Foxp3(+) T regulatory cell function and promotes antitumor immunity. Nat Med.

[49] Song L, Gao M, Dong W, Hu M, Li J, Shi X, Hao Y, Li Y, Huang C (2011). p85alpha mediates p53 K370 acetylation by p300 and regulates its promoter-specific transactivity in the cellular UVB response. Oncogene.

